# Supplementary material for: Tryptophan-galactosylamine conjugates inhibit and disaggregate amyloid fibrils of Aβ42 and hIAPP peptides while reducing their toxicity
Source: Commun Biol. 2020 Sep 2;3:484. doi: 10.1038/s42003-020-01216-5 (PMC7468108; doi:10.1038/s42003-020-01216-5)
Supplement: Supplementary file 1 — Supplementary Information [file 42003_2020_1216_MOESM1_ESM.pdf]

# Supplementary Information

## Tryptophan-galactosylamine conjugates inhibit and disaggregate amyloid fibrils of Aβ42 and hIAPP peptides while reducing their toxicity

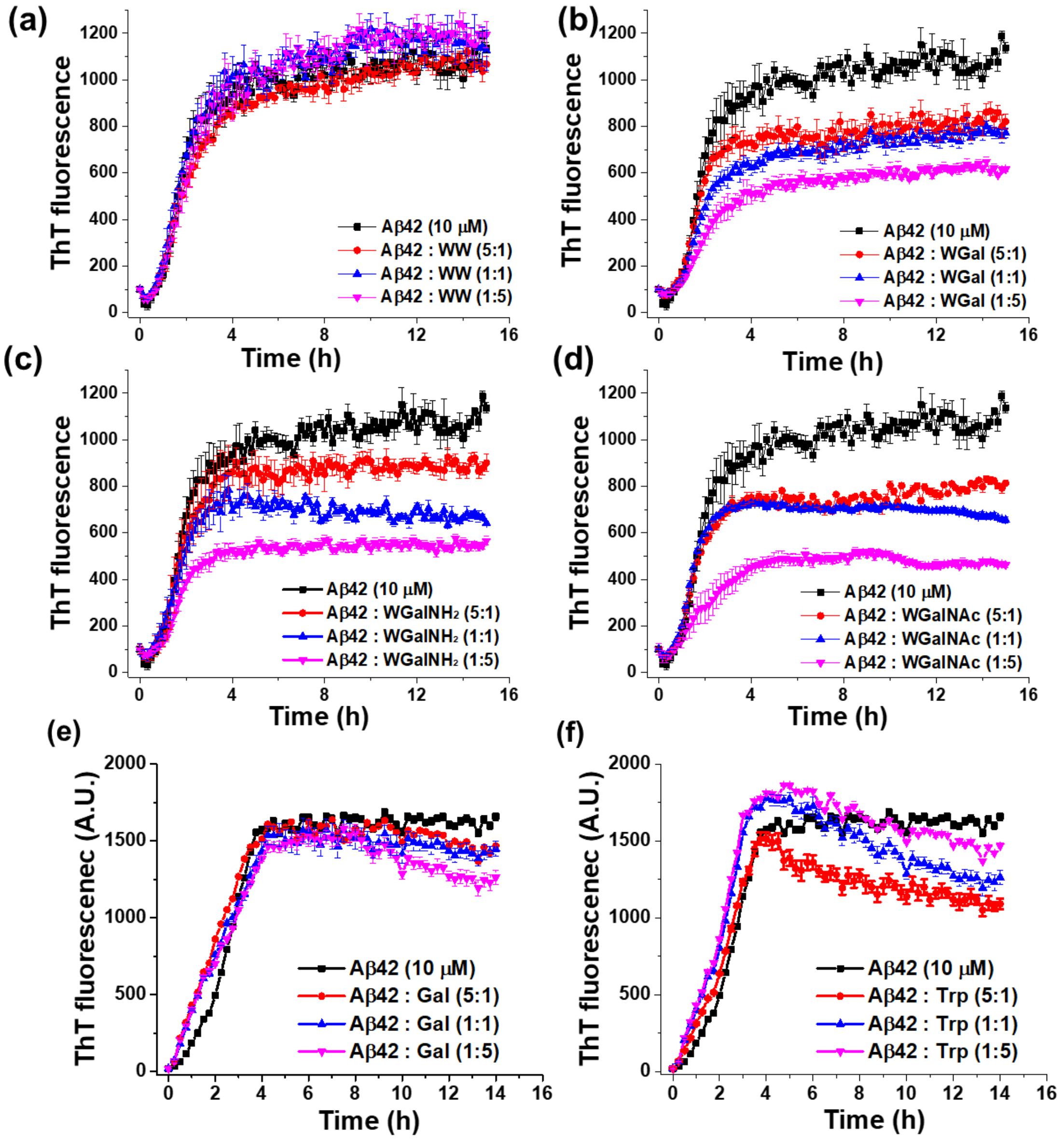


**Supplementary Figure 1. ThT-binding assay of Aβ42 in the presence of the tryptophan-galactosylamine hybrid molecules.** Black: Aβ42:hybrid molecule=1:0, red: Abeta42:hybrid molecule=5:1; blue: Aβ42:hybrid molecule=1:1; pink: Abeta42:hybrid molecule=1:5. **a.** WW; **b.** WGal; **c.** $WGalNH_2$; **d.** WGalNAc; **e.** galactosylamine (gal); **f.** tryptophan (Trp).

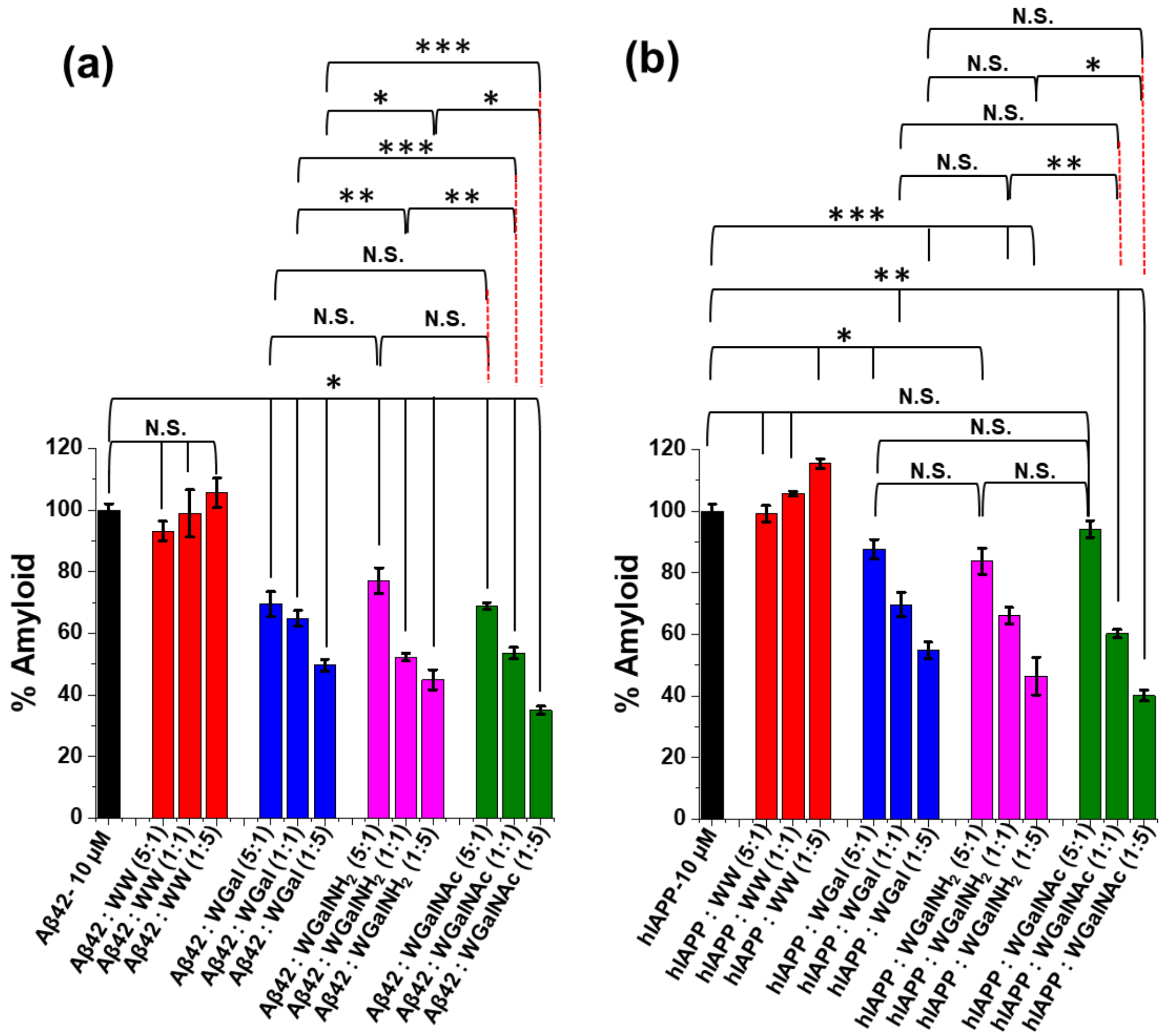


**Supplementary Figure 2. Percentage of amyloid formation and statistical analysis of the effect of the tested molecules on aggregation of Aβ42 and hIAPP. (a)** Statistical significance and *p*-values of the inhibitory effect of the hybrid molecules compared to Aβ42 alone and compared to each other; **(b)** Statistical significance and *p*-values of the inhibitory effect of the hybrid molecules compared to hIAPP alone and compared to each other. The percentage of amyloid formation was calculated the end of the ThT-binding assays. N.S.: not significant; *: $p$-value < 0.05; **: $p$-value < 0.005; ***: $p$-value < 0.001.

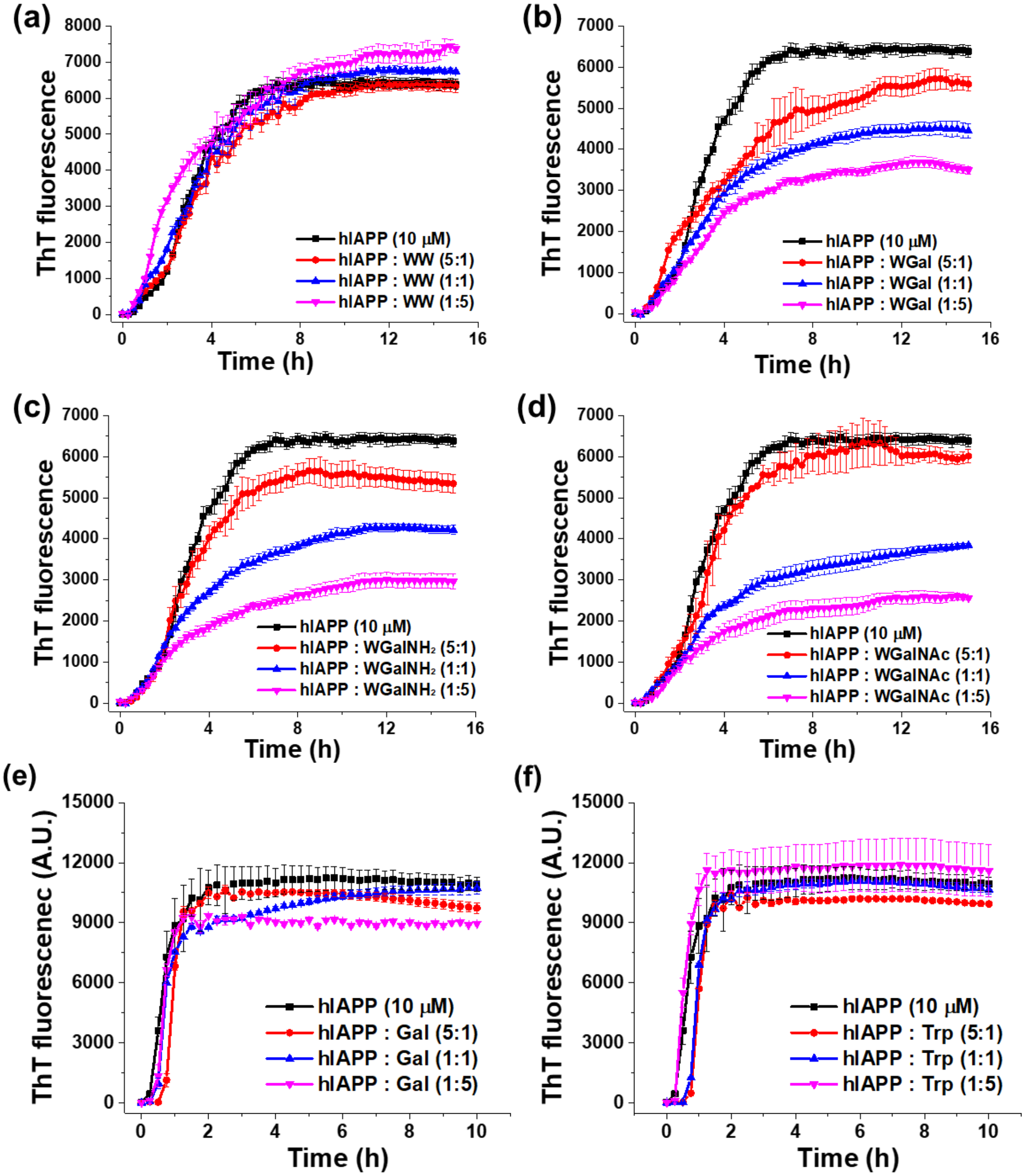


**Supplementary Figure 3. ThT-binding assay of hIAPP in the presence of the tryptophan-galactosylamine hybrid molecules.** Black: hIAPP:hybrid molecule=1:0, red: hIAPP:hybrid molecule=5:1; blue: hIAPP:hybrid molecule=1:1; pink: hIAPP:hybrid molecule=1:5. **a.** WW; **b.** WGal; **c.** $WGalNH_2$; **d.** WGalNAc; **e.** galactosylamine (gal); **f.** tryptophan (Trp).

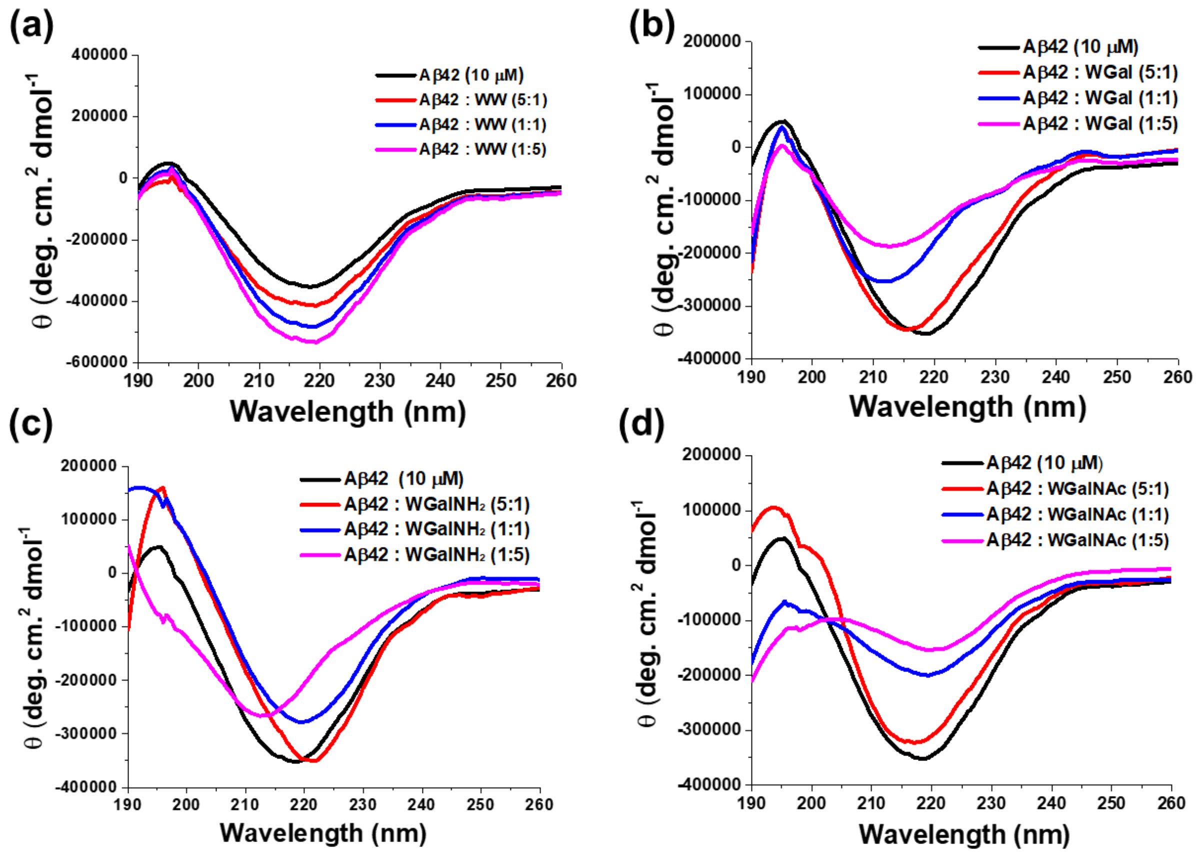


**Supplementary Figure 4. CD analysis of aggregated Aβ42 in the presence of the tryptophan-galactosylamine hybrid molecules.** Black: Aβ42:hybrid molecule=1:0, red: Aβ42:hybrid molecule=5:1; blue: Aβ42:hybrid molecule=1:1; pink: Aβ42:hybrid molecule=1:5. **a.** WW; **b.** WGal; **c.** WGalNH$_2$; **d.** WGalNAc

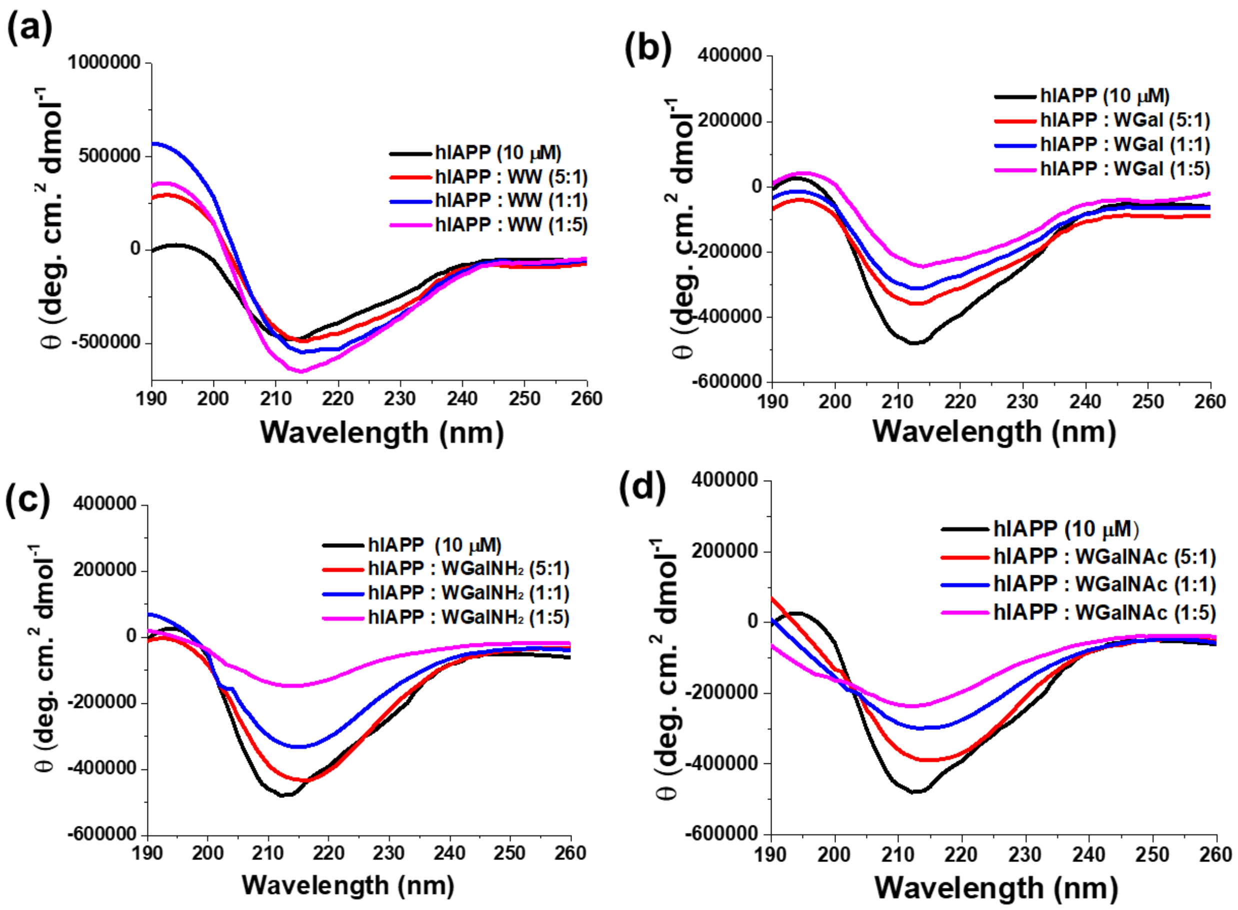


**Supplementary Figure 5. CD analysis of aggregated hIAPP in the presence of the tryptophan-galactosylamine hybrid molecules.** Black: hIAPP:hybrid molecule=1:0, red: hIAPP:hybrid molecule=5:1; blue: hIAPP:hybrid molecule=1:1; pink: hIAPP:hybrid molecule=1:5. **a.** WW; **b.** WGal; **c.** WGalNH$_2$; **d.** WGalNAc.

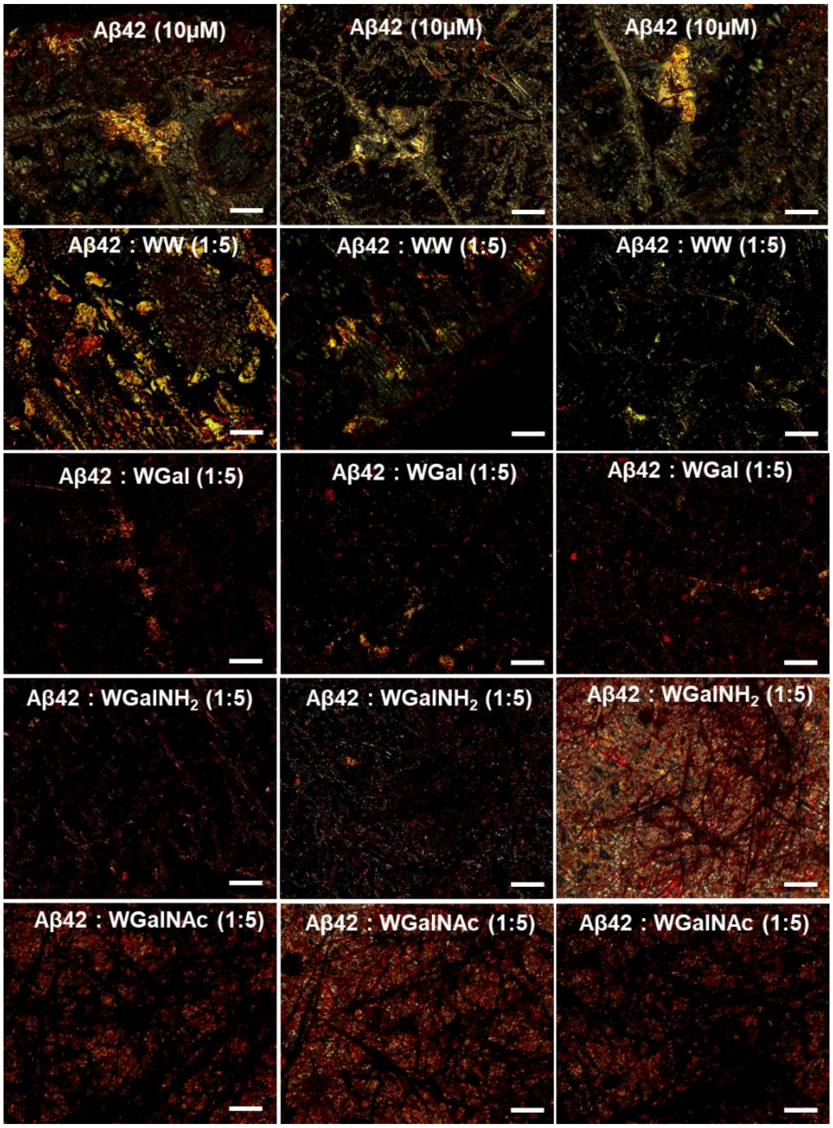


**Supplementary Figure 6. Analysis of Aβ42 fibrils in the absence or presence of the tryptophan-galactosylamine hybrid molecules.** Additional Congo red stained birefringence representative images of Aβ42 fibrils in the absence or presence of the 5-fold molar excess of the tested molecules. All scale bars, 100 μm.

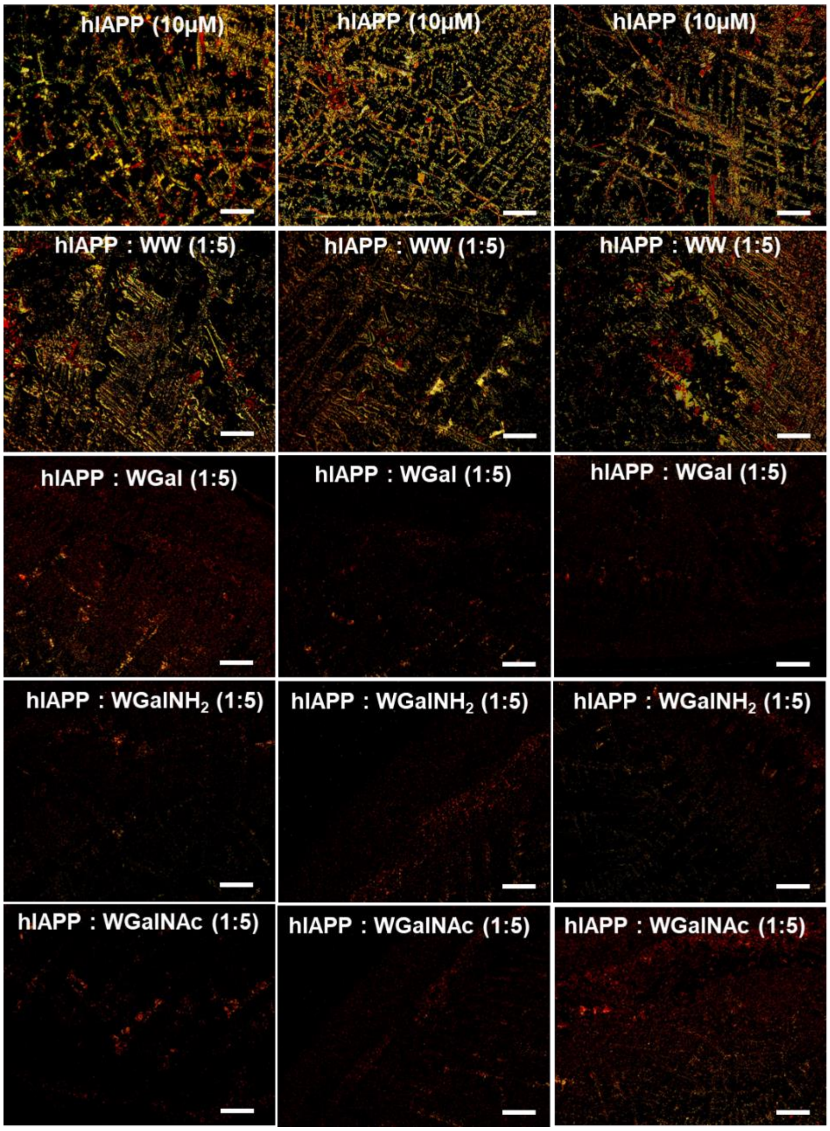


**Supplementary Figure 7. Analysis of hIAPP fibrils in the absence or presence of the tryptophan-galactosylamine hybrid molecules.** Additional Congo red stained birefringence representative images of hIAPP fibrils in the absence or presence of the 5-fold molar excess of the tested molecules. All scale bars, 100 μm.

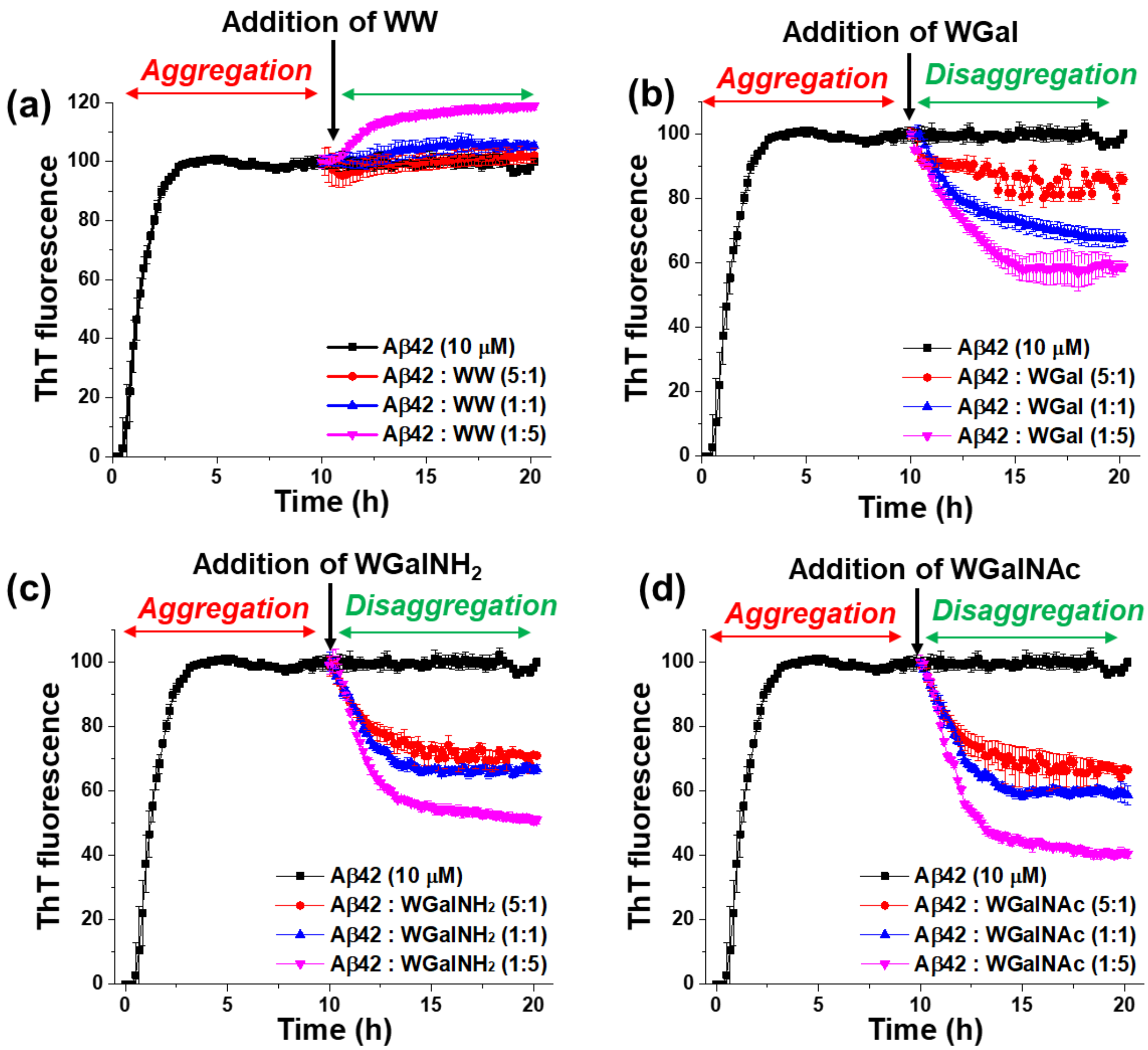


**Supplementary Figure 8. ThT kinetics for the disaggregation of preformed Aβ42 fibrils in the presence of the tryptophan-galactosylamine hybrid molecules.** Black: Aβ42:hybrid molecule=1:0, red: Aβ42:hybrid molecule=5:1; blue: Aβ42:hybrid molecule=1:1; pink: Aβ42:hybrid molecule=1:5. **a.** WW; **b.** WGal; **c.** WGalNH$_2$; **d.** WGalNAc.

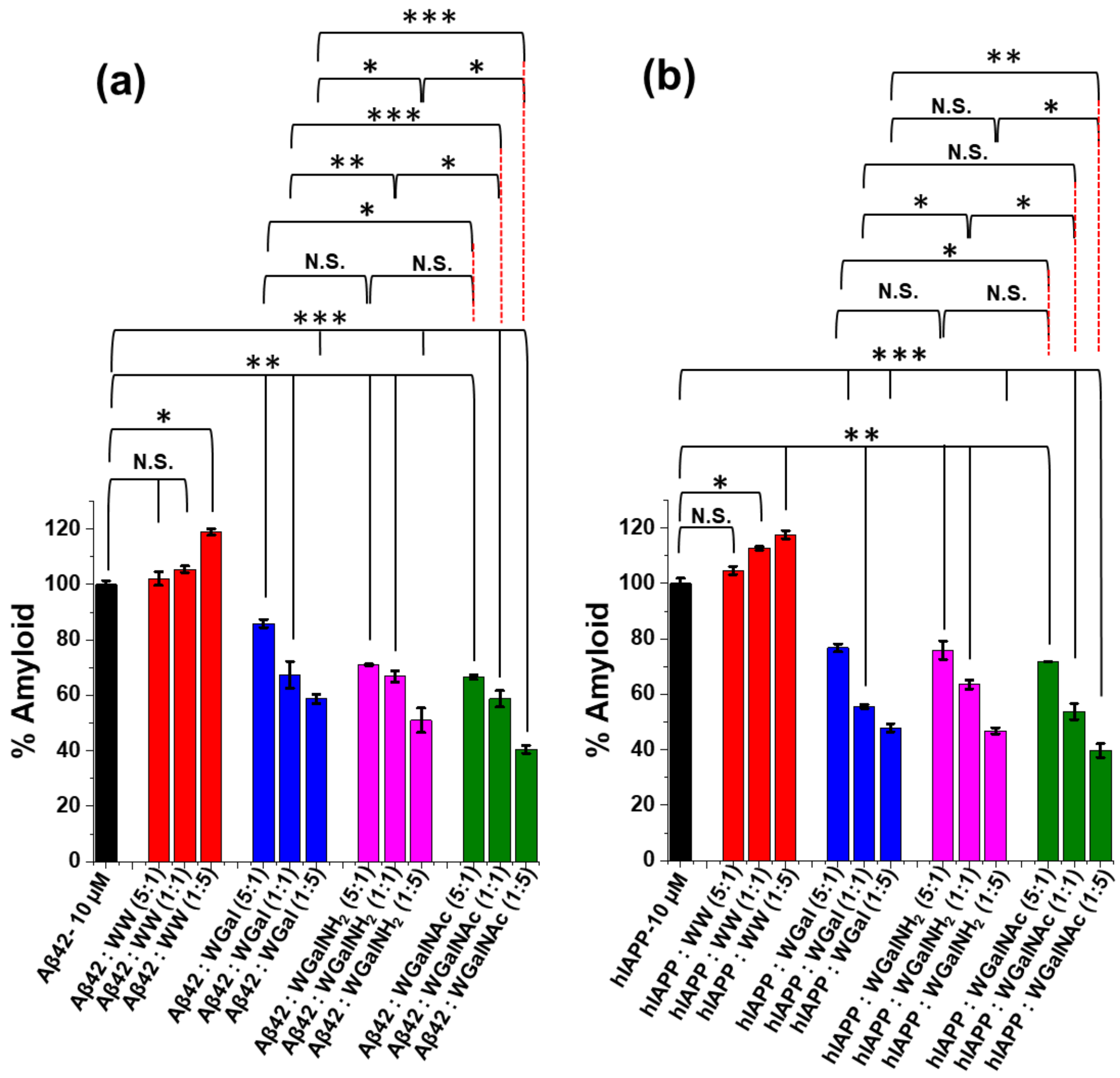


**Supplementary Figure 9. Percentage of amyloid disruption and statistical analysis of the effect of the tested molecules on the disruption of pre-formed Aβ42 and hIAPP aggregates.** **(a)** Statistical significance and *p*-values of Aβ42 aggregate disruption by the hybrid molecules compared to Aβ42 alone and compared to each other; **(b)** Statistical significance and *p*-values of hIAPP aggregate disruption by the hybrid molecules compared to hIAPP alone and compared to each other. The percentage of pre-formed amyloid disruption was calculated at the end of the ThT-binding assays. N.S.: not significant; *: *p*-value < 0.05; **: *p*-value < 0.005; ***: *p*-value < 0.001.

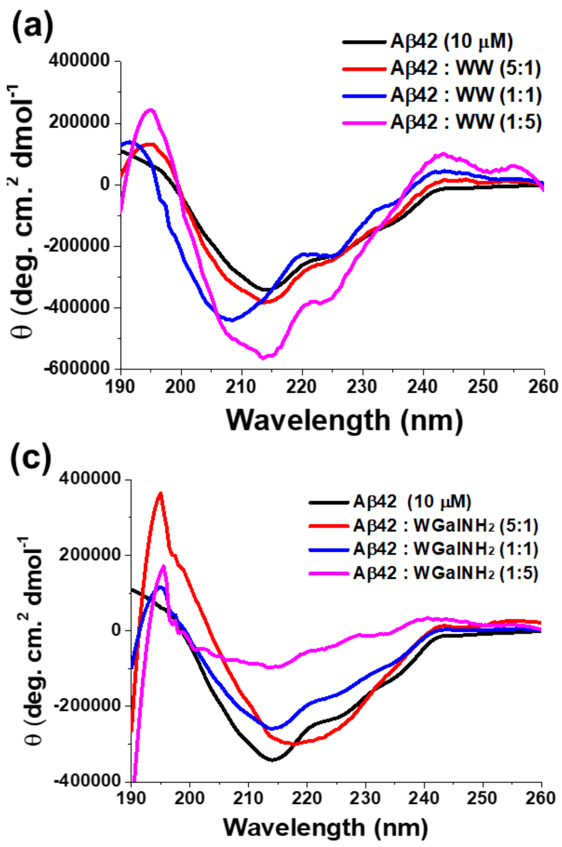


**Supplementary Figure 10. CD analysis of disaggregated Aβ42 preformed fibrils in the presence of the tryptophan-galactosylamine hybrid molecules.** Black: Aβ42:hybrid molecule=1:0, red: Aβ42:hybrid molecule=5:1; blue: Aβ42:hybrid molecule=1:1; pink: Aβ42:hybrid molecule=1:5. **a.** WW; **b.** WGal; **c.** WGalNH$_2$; **d.** WGalNAc.

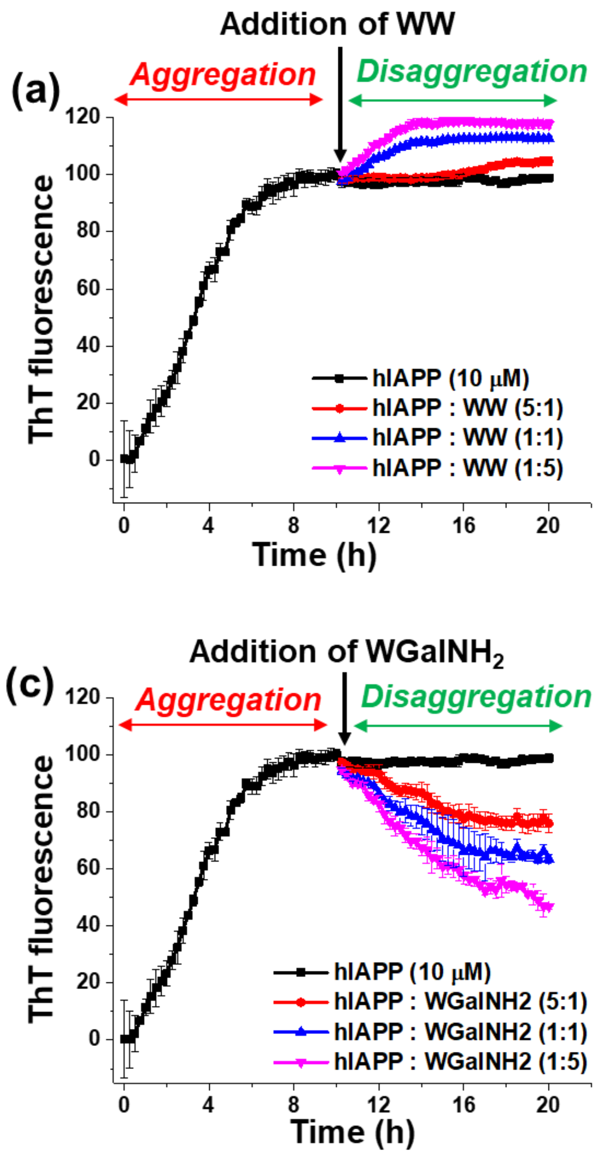


**Supplementary Figure 11. ThT kinetics for the disaggregation of preformed hIAPP fibrils in the presence of the tryptophan-galactosylamine hybrid molecules.** Black: hIAPP:hybrid molecule=1:0, red: hIAPP:hybrid molecule=5:1; blue: hIAPP:hybrid molecule=1:1; pink: hIAPP:hybrid molecule=1:5. **a.** WW; **b.** WGal; **c.** $WGalNH_2$; **d.** WGalNAc.

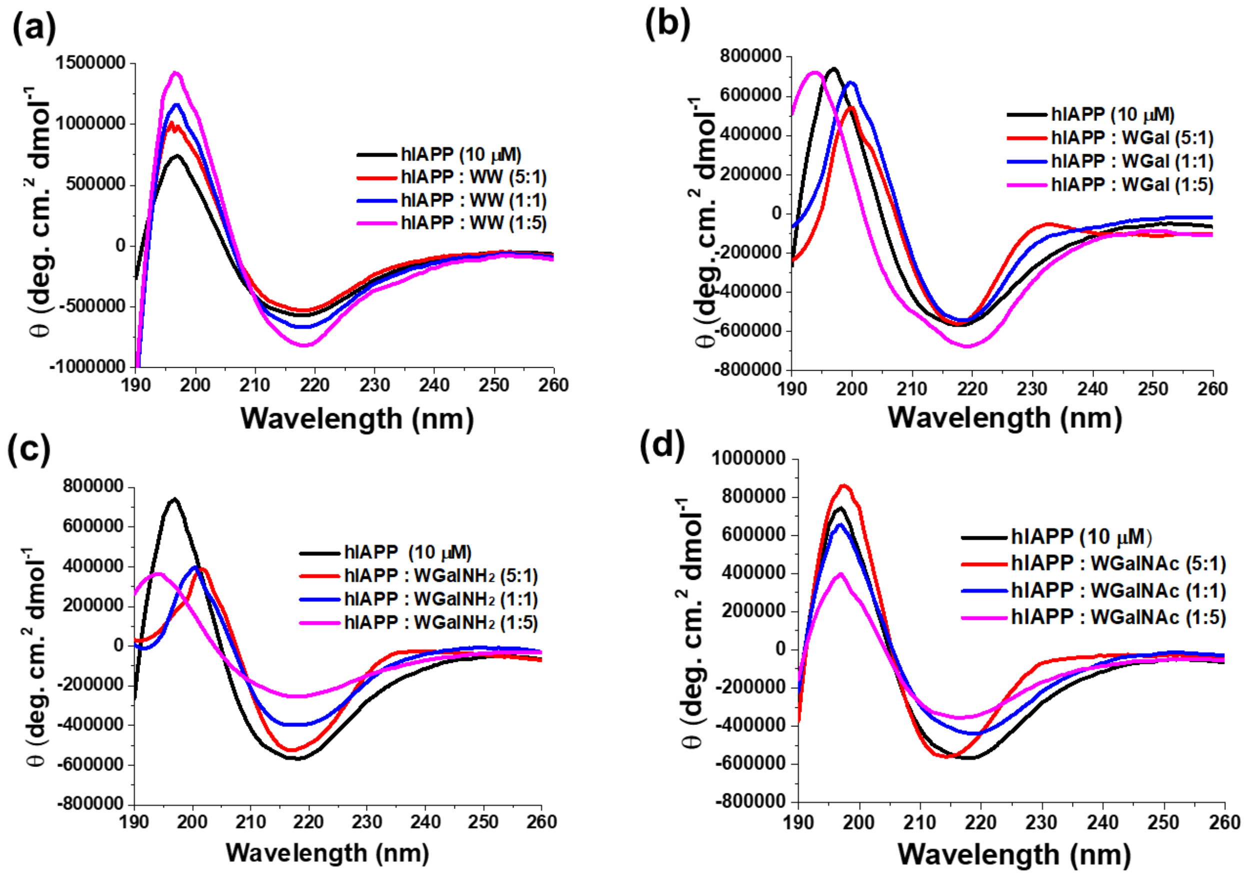


**Supplementary Figure 12. CD analysis of disaggregated hIAPP preformed fibrils in the presence of the tryptophan-galactosylamine hybrid molecules.** Black: hIAPP:hybrid molecule=1:0, red: hIAPP:hybrid molecule=5:1; blue: hIAPP:hybrid molecule=1:1; pink: hIAPP:hybrid molecule=1:5. **a.** WW; **b.** WGal; **c.** WGalNH$_2$; **d.** WGalNAc.

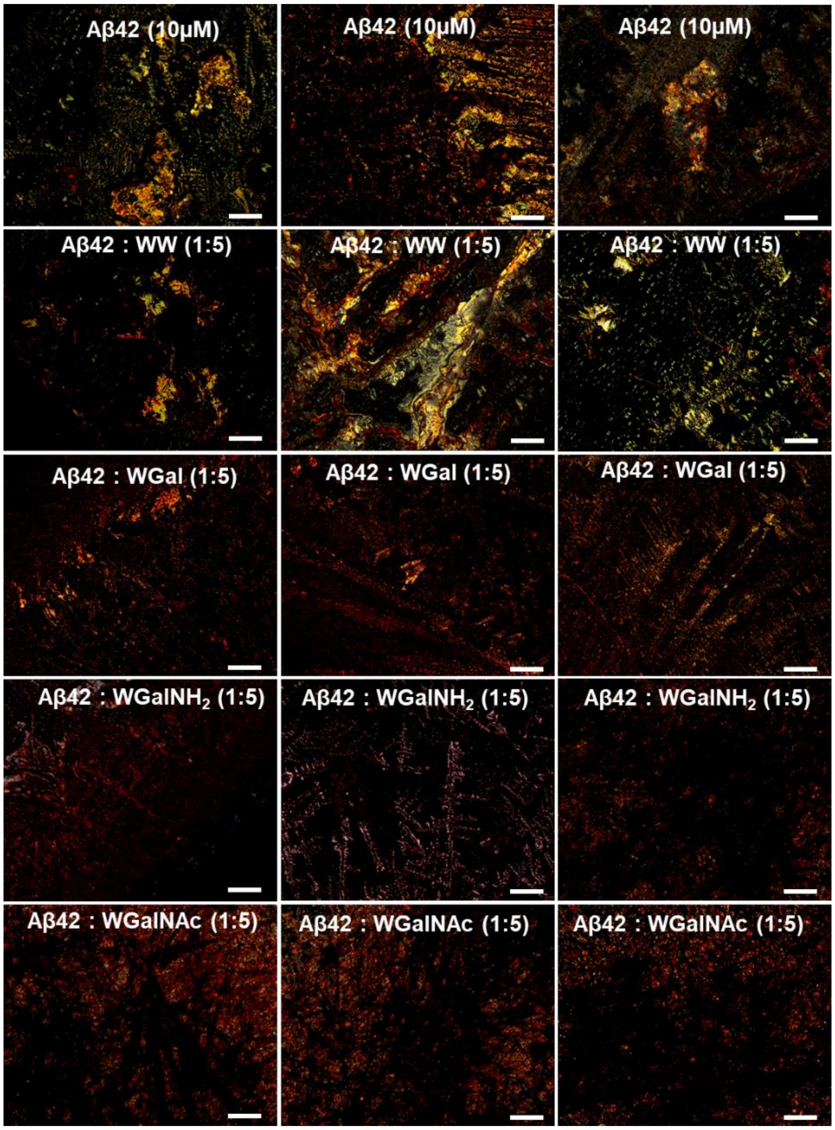


**Supplementary Figure 13. Analysis of pre-formed Aβ42 fibrils in the absence or presence of the tryptophan-galactosylamine hybrids.** Additional Congo red stained birefringence representative images of Aβ42 fibrils in the absence or presence of the 5-fold molar excess of the tested molecules. All scale bars, 100 μm.

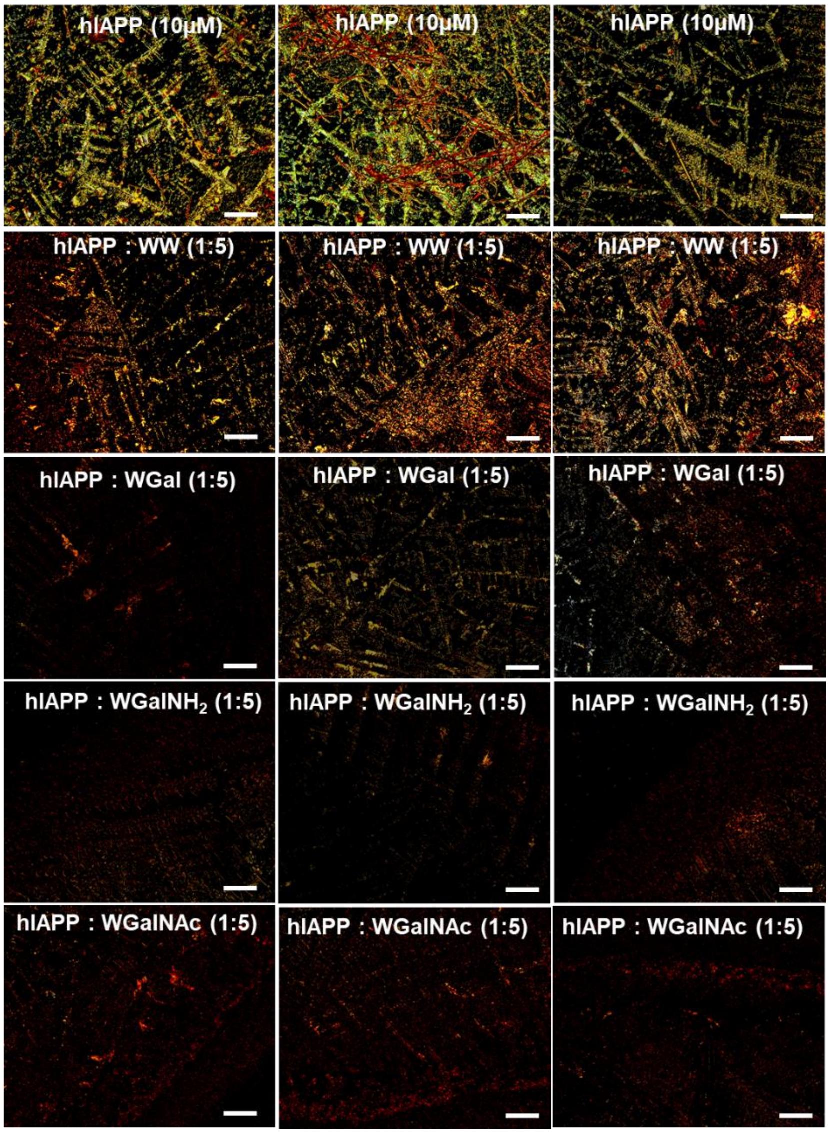


**Supplementary Figure 14. Analysis of pre-formed hIAPP fibrils in the absence or presence of the tryptophan-galactosylamine hybrids.** Additional Congo red stained birefringence representative images of hIAPP fibrils in the absence or presence of the 5-fold molar excess of the tested molecules. All scale bars, 100 μm.

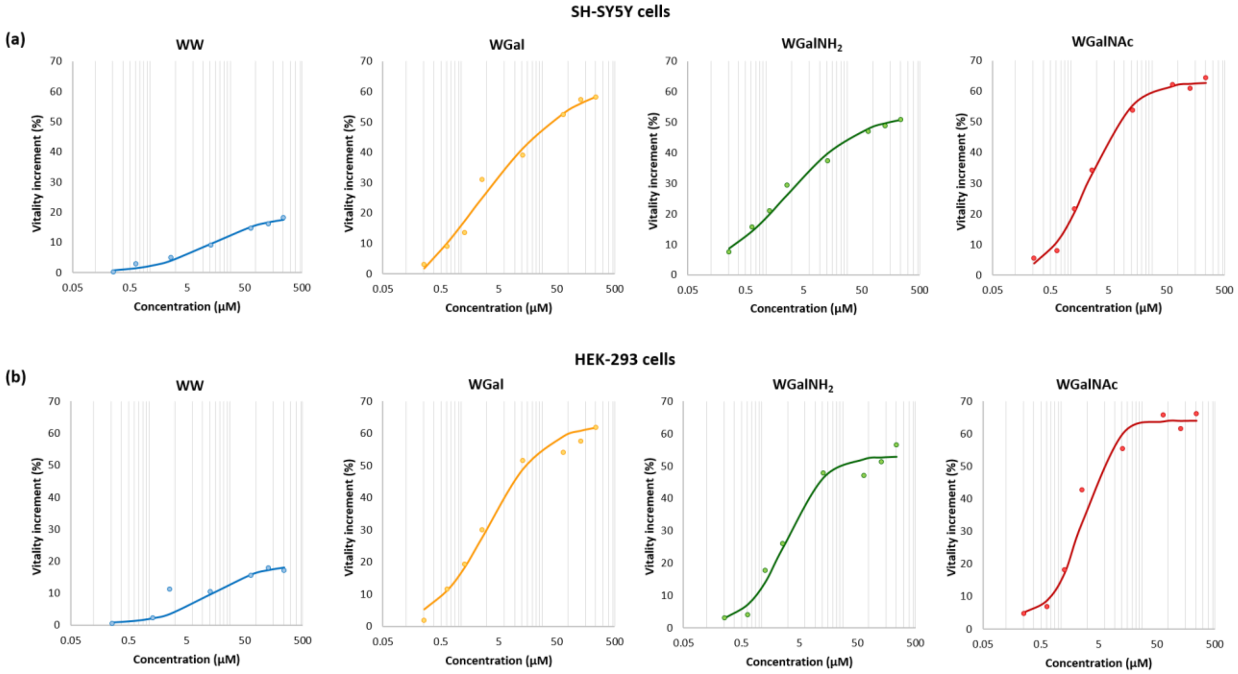


**Supplementary Figure 15. Dose-response curves of cell viability increment of calculated from XTT assay (Figure 9). (a)** Increment cell viability (%) of SH-SY5Y upon treatment of different concentrations of the tested molecules on Aβ42 (13 µM) preformed fibrils and **(b)** Increment cell viability (%) of HEK-293 cells upon treatment of different concentrations of the tested molecules on hIAPP (10 µM) preformed fibrils.
