# Supplementary material for: Tryptophan-galactosylamine conjugates inhibit and disaggregate amyloid fibrils of Aβ42 and hIAPP peptides while reducing their toxicity
Source: Commun Biol. 2020 Sep 2;3:484. doi: 10.1038/s42003-020-01216-5 (PMC7468108; doi:10.1038/s42003-020-01216-5)
Supplement: Supplementary file 2 — Description of Additional Supplementary Files [file 42003_2020_1216_MOESM2_ESM.pdf]

Description of Additional Supplementary Files

File Name: Supplementary Data 1
Description: Source data for Figures 2, 5, 8, 9 and 10
